# APOBEC3B and APOBEC mutational signature as potential predictive markers for immunotherapy response in non-small cell lung cancer

**DOI:** 10.1038/s41388-018-0245-9

**Published:** 2018-04-26

**Authors:** Shixiang Wang, Mingming Jia, Zaoke He, Xue-Song Liu

**Affiliations:** 1 School of Life Science and Technology, ShanghaiTech University, Shanghai, 201203 China; 2 Shanghai Institute of Biochemistry and Cell Biology, Chinese Academy of Sciences, Shanghai, China; 3 University of Chinese Academy of Sciences, Beijing, China

## Abstract

Non-small cell lung cancer (NSCLC) is known to carry heavy mutation load. Besides smoking, cytidine deaminase APOBEC3B plays a key role in the mutation process of NSCLC. APOBEC3B is also reported to be upregulated and predicts bad prognosis in NSCLC. However, targeting APOBEC3B high NSCLC is still a big challenge. Here we show that APOBEC3B upregulation is significantly associated with immune gene expression, and APOBEC3B expression positively correlates with known immunotherapy response biomarkers, including: PD-L1 expression and T-cell infiltration in NSCLC. Importantly, APOBEC mutational signature is specifically enriched in NSCLC patients with durable clinical benefit after immunotherapy and APOBEC mutation count can be better than total mutation in predicting immunotherapy response. In together, this work provides evidence that APOBEC3B upregulation and APOBEC mutation count can be used as novel predictive markers in guiding NSCLC checkpoint blockade immunotherapy.

## Introduction

Lung cancer is the leading cause of cancer death worldwide, and the two main types are small cell lung cancer (SCLC) and non-SCLC (NSCLC). About 80–85% of lung cancers are NSCLC, and about 10–15% are SCLC. The three main subtypes of NSCLC are adenocarcinoma, squamous cell carcinoma and large-cell carcinoma [1]. NSCLC is known for its heavy mutation load, and smoking is one major cause of the heavy mutation load of NSCLC [2]. Besides smoking, expression of APOBEC family members, especially APOBEC3B was reported as a key source of mutations in NSCLC [3].

The APOBEC family of zinc-coordinating enzymes convert cytosines to uracils (C-to-U) in single-strand DNA. The enzymatic activity of this family member is essential for both adaptive and innate immune responses [4]. Notably, APOBEC3B is upregulated, and its preferred target sequence is frequently mutated and clustered in several cancers especially NSCLC [3]. Tobacco smoking-related mutations appear to have a strong role in tumor initiation, whereas APOBEC-related mutations are more prominent at a later stage of NSCLC development and are associated with tumor progression and metastasis [5, 6].

Although it is known that APOBEC3B plays a critical role in NSCLC, targeting APOBEC3B overexpressed NSCLC is still a big challenge. It was assumed to suppress cancer progression through inhibiting APOBEC3B expression. Currently, there are no available drugs that can inhibit APOBEC3B expression or function. Traditionally, NSCLC is treated by radiation, surgery and chemical therapy. Recently, immunotherapy with antibodies targeting immune checkpoints programmed cell death protein-1 (PD-1) and ligand (PD-L1) signaling have been approved for the treatment of human cancers [7–12]. In advanced NSCLC, therapies with an antibody targeting PD-1 demonstrated response rates of 17–21%, with some responses being remarkably durable [8]. Although clinical studies have shown promise for targeting PD-1, PD-L1 signaling in NSCLC, the factors that predict which patients will be responsive to checkpoint blockade are not fully understood.

Here we report through systematic cancer genomics and transcriptomic association studies that APOBEC3B overexpression is associated with immune gene expression and known immunotherapy response biomarkers, APOBEC mutational signature is specifically enriched in patients with durable clinical benefit (DCB) after immunotherapy and APOBEC mutation count can be better than total mutation count in predicting immunotherapy response. Our study implicates APOBEC3B and APOBEC mutational signature as novel predictive biomarkers for checkpoint blockade immunotherapy response in NSCLC.

## Results

### APOBEC3B expression and mutational signature in NSCLC

**Fig. 1:**
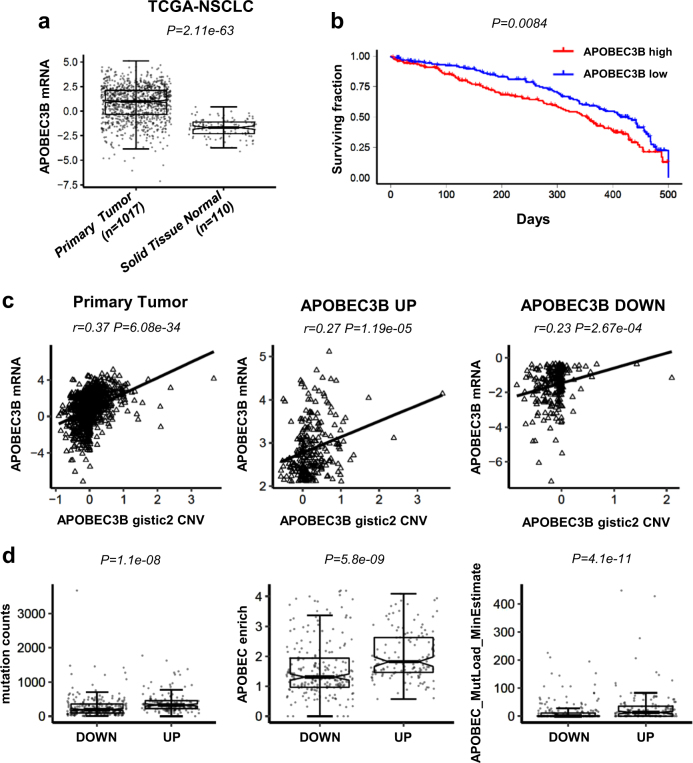
*APOBEC3B* expression, prognosis and mutational signature in non-small cell lung cancer (NSCLC). **a** *APOBEC3B* mRNA expression in NSCLC primary tumors (*n* = 1017) and normal control (*n* = 110) lung tissues are shown. The values of mRNA expression are log2 based normalized count. **b** Kaplan–Meier overall survival curve of NSCLC patients are shown. Patients are separated into two groups based on *APOBEC3B* mRNA expression. APOBEC3B high (*n* = 190 with matched survival information) was defined as samples with *APOBEC3B* mRNA expression values above the population mean, whereas the remaining samples were defined as APOBEC3B-low (*n* = 172). **c** Correlations between *APOBEC3B* copy number and mRNA expression in NSCLC (*n* = 1017). APOBEC3B-UP (*n* = 254) was defined as samples with *APOBEC3B* mRNA expression values above the third quartile, whereas APOBEC3B-DOWN (*n* = 254) was defined as samples with *APOBEC3B* mRNA expression values below the first quartile. **d** Mutation counts (left panel) and enrichment values of the APOBEC mutagenesis signature (middle and right panel) between APOBEC3B-UP (133 samples with matched mutation information) and DOWN (215 samples matched) NSCLC samples. The boxplot is bounded by the first and third quartile with a horizontal line at the median. The notch shows the 95% confidence interval of the median

NSCLC is a leading cause of death worldwide. Its typical features include heavy mutation load, and cytidine deaminase APOBEC3B has been implicated as an important source of mutation in NSCLC [3, 13]. APOBEC3B-related mutational process fuels cancer evolution and treatment resistance, and still remains a big challenge for NSCLC treatment. *APOBEC3B* expression was reported to be upregulated in lung cancer [3, 14], and this is independently verified in NSCLC with TCGA datasets (Fig. 1a). However, how APOBEC3B expression is regulated during cancer evolution still remain elusive. Our analysis based on public TCGA database implicates *APOBEC3B* copy number variation (CNV) is amplified (gistic2 thresholded CNV > = 1 or gistic2 CNV > = 0.1) in 29.9% (304 of 1017) NSCLC samples, and *APOBEC3B* CNV significantly positively correlates with *APOBEC3B* mRNA (Fig. 1c), implicating CNV amplification is one driving force for *APOBEC3B* mRNA upregulation during cancer evolution. *APOBEC3B* CNV is usually co-amplified with the whole *APOBEC3* locus.

Increased APOBEC3B predicts worse outcomes in lung cancer [15]. This was also verified with TCGA NSCLC datasets (Fig. 1b). Probably *APOBEC3B* expression stimulates mutation process, and accelerates tumor evolution speed, and thus leads to bad prognosis for APOBEC3B high NSCLC. We further checked mutation load and APOBEC mutational signature in NSCLC. Total mutation counts are significantly upregulated in APOBEC3B UP patients compared with APOBEC3B DOWN patients (Fig. 1d). As expected APOBEC3B UP also leads to significantly increased APOBEC-related mutations (Fig. 1d).

### Association between *APOBEC3B* expression and immune gene expression signature in NSCLC

**Fig. 2:**
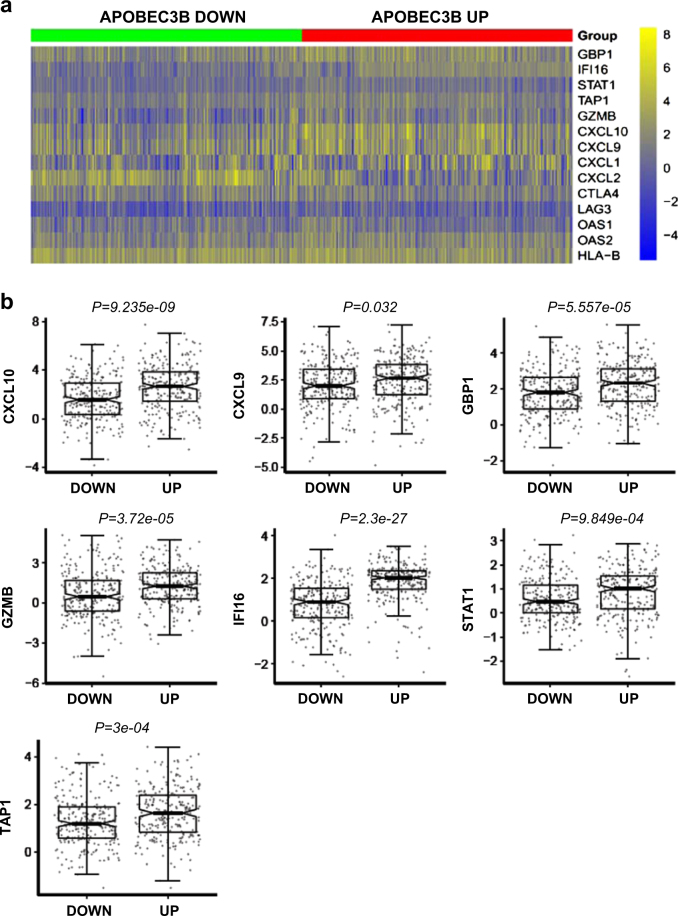
*APOBEC3B* upregulation is associated with T-effector and interferon-γ (IFN-γ) gene signature. **a** Heatmap depicting mRNA expression levels of genes in T-effector and interferon-γ (IFN-γ) gene signature. **b** Quantitative analysis of the genes in T-effector and IFN-γ gene signature based on *APOBEC3B* expression status. Both APOBEC3B-UP and APOBEC3B-DOWN group contain 254 NSCLC samples. Unpaired Student's* t*-test was performed and FDR adjusted *P*-values are shown

Due to the critical role of APOBEC3B in lung cancer progression and prognosis, we investigated the functional correlation of *APOBEC3B* expression in both NSCLC patient samples and lung cancer cell lines. The specifically enriched signaling pathway in APOBEC3B-overexpressing samples can be surrogate targets for APOBEC3B. Samples were separated into two groups based on *APOBEC3B* expression. Gene set enrichment analysis (GSEA) was performed on significantly different expressing genes between these two groups. Immune response-related gene sets are among top enriched gene signatures when compared APOBEC3B up with APOBEC3B down samples (Supplementary Figures S1a and b). These immune response-related gene signatures include: “Hallmark interferon gamma response” (Supplementary Figure S1a). The enrichment of immune signature in both NSCLC patient samples and lung cancer cell lines implicates a cancer cell intrinsic mechanism for the association between APOBEC3B and immune response gene expression. Genes included in these immune response signatures contain *STAT1*, *CXCL10*, *CXCL9* (Fig. 2). Interestingly, the pretreatment expression of these immune genes has been shown as predictive factor in cancer Immunotherapy [16].

### Association between *APOBEC3B* expression and known immunotherapy predictive biomarkers in NSCLC

**Fig. 3:**
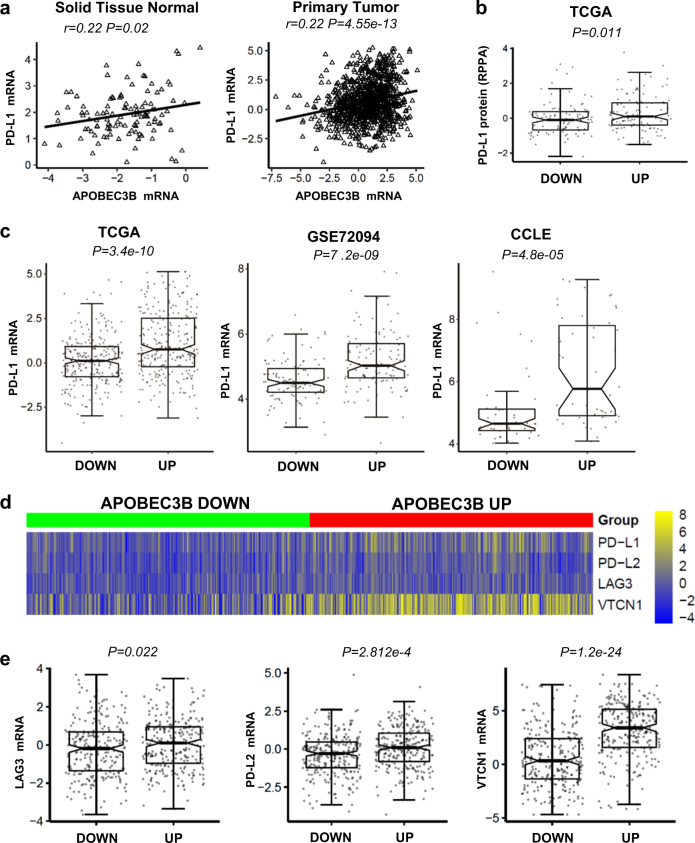
APOBEC3B expression positively correlates with PD-L1 and other immune checkpoint genes expression in NSCLC. **a** Correlations between *APOBEC3B* and *PD-L1* mRNA in normal lung tissue (*n* = 110) and NSCLC samples (*n* = 1017) are shown. **b** PD-L1 protein expression levels were compared between APOBEC3B UP (112 samples with matched PD-L1 protein expression data) and APOBEC3B DOWN (99 samples matched) groups based on TCGA datasets. **c** *PD-L1* mRNA expression levels were compared between APOBEC3B UP and APOBEC3B DOWN groups based on two NSCLC datasets, TCGA (*n* = 254 for both UP and DOWN group) and GSE72094 (*n* = 111 for both UP and DOWN group) and Cancer Cell Line Encyclopedia (CCLE) dataset (*n* = 47 for both UP and DOWN group). **d** Heatmap representation of relative mRNA expression levels of selected immune inhibitory checkpoints. **e** Quantitative analysis of immune checkpoints gene mRNA expression based on *APOBEC3B* expression status (*n* = 254 for both UP and DOWN group). Unpaired Student's *t-*test was performed and FDR adjusted *P*-values are shown

PD-L1 and PD-1 antibody have already been approved for treating NSCLC. But clinical predictors of response to these therapies remain incompletely characterized. Currently known biomarkers of response to anti‑PD-1/PD-L1 therapies include: PD-L1 expression [17, 18], tumor mutational load [19, 20], DNA mismatch repair (MMR) deficiency [21] and CD8^+^ T-cell intensity [22, 23]. We observed a significant positive correlation between *APOBEC3B* expression and *PD-L1* mRNA expression in NSCLC samples (Fig. 3a). APOBEC3B upregulation also leads to significantly increased PD-L1 protein expression (Fig. 3b). And this positive association between APOBEC3B and PD-L1 has been confirmed in different NSCLC databases GSE72094, and also lung cancer cells lines Cancer Cell Line Encyclopedia (CCLE) database. This further implicates cancer cell intrinsic mechanism for *APOBEC3B* and *PD-L1* expression correlation (Fig. 3c). In addition, a significant correlation between *APOBEC3B* expression and genes encoding other immune checkpoints, including *PD-L2* was also observed (Figs. 3d, e).

**Fig. 4:**
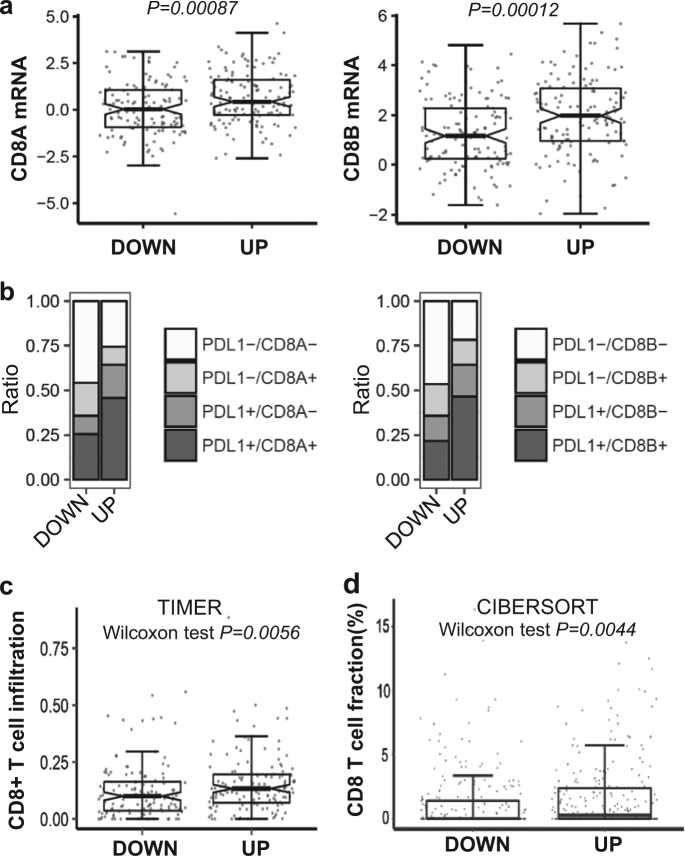
*APOBEC3B* upregulation is associated with increased CD8^+^ T-cell infiltration in NSCLC. **a** *CD8A* and *CD8B* mRNA expressions were compared between APOBEC3B UP and APOBEC3B DOWN groups based on TCGA dataset. **b** The correlation between *APOBEC3B* expression status and tumor microenvironment immune types classified based on PD-L1 and CD8 expression. Positive PD-L1 and CD8 were defined as above-median expression. **c** **and d** Abundance of CD8^+^ immune cell infiltration was quantified based on mRNA expression data with TIMER (**c**) or CIBERSORT (**d**) (Wilcoxon test, details see methods). *n* = 254 for both APOBEC3B-UP and APOBEC3B-DOWN group

APOBEC3B upregulation was found to be associated with significantly increased CD8A and CD8B expression (Figs. 4a, b). CD8^+^ T-cell infiltration levels can be quantified based on mRNA expression [24]. Significantly increased CD8^+^ T-cell infiltration was observed in APOBEC3B UP compared with APOBEC3B DOWN NSCLC samples (Fig. 4c, d). In together, our analysis indicates *APOBEC3B* expression positively correlates with PD-L1 expression and CD8^+^ T-cell infiltration, two known immunotherapy predictive biomarkers, suggests that *APOBEC3B* expression itself can be served as a novel predictor for immunotherapy response.

### Specific enrichment of APOBEC mutational signature in NSCLC with good immune therapy response

Analyses of tumor-derived genome sequences have shown that APOBEC over-activation leads to distinct patterns of base-substitution mutations termed signature 2 and signature 13 [25, 26]. These mutation patterns, among others, can be recognized via non-negative matrix factorization (NMF), a technique used to identify recurring patterns (i.e., signatures) in the spectra of mutations from a set of tumors and to estimate the contributions of these signatures to the mutational landscape of the individual tumors [26]. It has been reported previously that total mutation load predicts immunotherapy response [20], but it was still not known if some types of mutation is better than other mutation types in predicting immunotherapy response. We then compare the mutational signatures between patients with DCB and patients with no durable benefit (NDB) after immunotherapy based on recently published NSCLC anti-PD-1 immune checkpoint blockade therapy dataset [20].

**Fig. 5:**
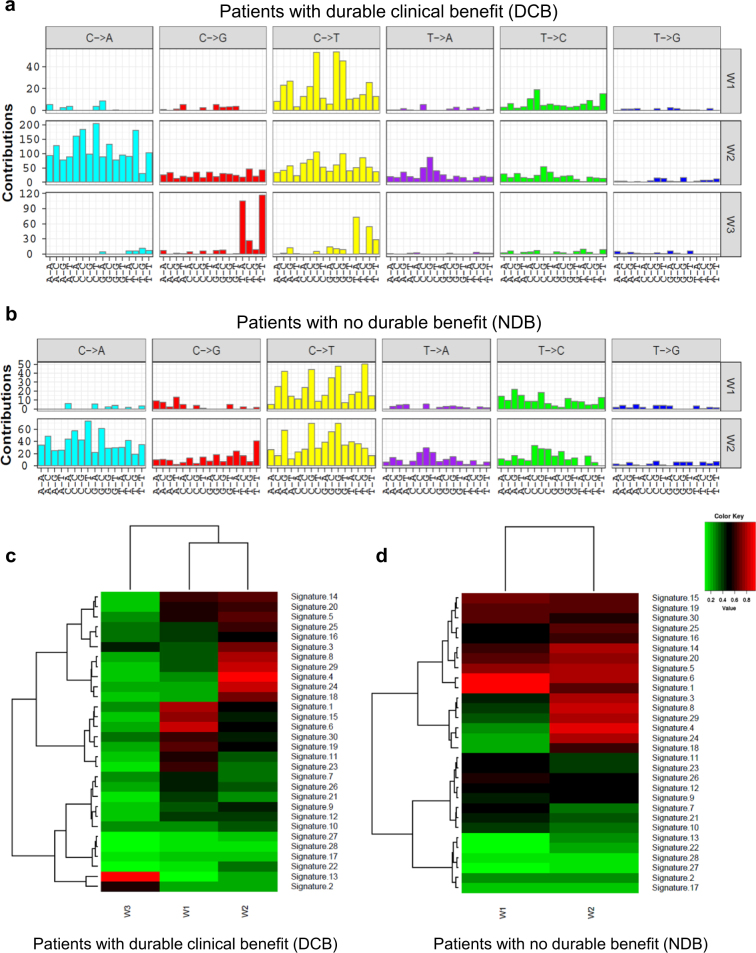
APOBEC mutational signature is specifically enriched in patients with durable clinical benefit (DCB, *n* = 14) but not patients with no durable benefit (NDB, *n* = 17). **a**,** b** A Bayesian NMF algorithm was applied to identify signatures from the matrix of mutation counts across NSCLC patients with DCB **a** or patients with NDB **b**. Three distinct mutational signatures were identified in DCB patients, whereas NDB samples contain two distinct mutational signatures. **c**, **d** Cosine similarity analysis of identified mutational signatures in NSCLC with DCB **c** or NSCLC with NDB **d**. The specifically identified W3 mutational signature in DCB patients is similar to previously reported mutational signature 2, 13, which are known to be caused by the action of APOBEC

In DCB patients, three mutational signatures are recurrently enriched in 100% simulations, whereas in 72% simulations two mutational signatures are enriched in NDB patients (Fig. 5 and Supplementary Figure S2). Mutational signature W3 is the distinct mutational signature specifically enriched in DCB patients (Fig. 5a, b). Cosine similarity analysis indicates that W3 is similar to previously reported mutational signature 2 and 13 (Fig. 5c, d). It is known that mutational signature 2 and 13 are caused by APOBEC family members [25, 26]. Thus, only DCB patients show enrichment of APOBEC mutational signature. The overlapping mutational signature W1, W2 in DCB and NDB patients are predicted to be caused majorly by DNA mismatch repair defect and smoking respectively (Fig. 5c, d) [26]. Interestingly, in 28% simulations only one mutational signature (W2, smoking signature) is enriched in NDB patients (Supplementary Figure S2), and this is in line with the fact that DNA mismatch repair (W1 mutational signature) is already approved as a specific marker for DCB patients [21]. In together, our study implicates for the first time that APOBEC mutational signature is specifically associated with patients with good immunotherapy response.

Pre-therapy immune gene expression has been shown to predict immunotherapy response [27]. We compared the difference between APOBEC signature mutation and other types of mutation in association with immune gene expression using 1000 TCGA NSCLC samples. Four mutational signatures are enriched in this dataset, named as W1 through W4 (Supplementary Figure S3a). Cosine similarity analysis indicate W4 signature is caused by APOBEC (Supplementary Figure S3b). These NSCLC samples were ranked from left to right based on the average Log2 expression of 60 immune genes [28], and the contribution of each mutational signatures to all the samples are listed below (Supplementary Figure S3c). Statistical analysis indicates only W4 signature (caused by APOBEC) mutation show significant (*P* = 0.04656) association with increased immune gene expression (Supplementary Figure S3d). This study supports our original observation that APOBEC mutation could be specific mutation type that are associated with immune gene expression and immune therapy response.

### APOBEC mutation count can be a novel predictive biomarker for immunotherapy efficacy

**Fig. 6:**
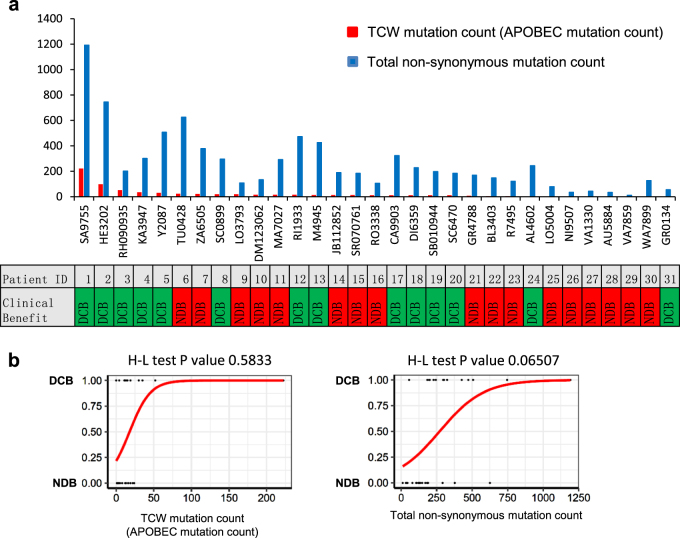
TCW mutation count (APOBEC mutation count) can be better than total non-synonymous mutation count in predicting PD-1 blockade immunotherapy clinical response. TCW (W = A or T) mutation count (including: TCA to TTA or TGA, TCT to TTT or TGT) can be a represent of APOBEC mutation count. **a** TCW mutation count and total non-synonymous mutation count of 31 NSCLC patients with durable clinical benefit (DCB, *n* = 14) or no durable benefit (NDB, *n* = 17) after immunotherapy are shown. **b** The goodness-of-fit were performed by Hosmer–Lemeshow test, which showed that the TCW mutation counts is more suitable for predicting prognosis of patients than total non-synonymous mutation counts

APOBEC activation in cancer leads to elevated levels of genomic C-to-U deamination events, which manifest as C-to-T transitions or C-to-G transversions within TCW (W = A or T) trinucleotide contexts [3]. Combined mutations in TCW context (including: TCA to TTA or TGA, TCT to TTT or TGT) can be a represent of APOBEC mutation count. To evaluate, the practical usage of APOBEC mutation count as a predictive biomarker for NSCLC immune therapy response. We calculate APOBEC mutation count in the exome sequencing dataset of NSCLC samples with durable or no DCB to immunotherapy (Fig. 6a). Average APOBEC mutation count in all NSCLC samples is 23 (*n* = 31, range 0–222). Interestingly, in all five patients (100%) with >24 APOBEC mutation count show DCB to immunotherapy. The average APOBEC mutation count of DCB patients is 38 (*n* = 14), compared with 10 of NDB patients (*n* = 17), is 2.8-fold higher. Average total mutation count is 268 (range 11–1192). Eight of 11 (72.7%) patients with more than average total mutation show DCB after immunotherapy. The average total mutation of DCB patients is 384, and is 1.3-fold higher than NDB patients.

Logistic regression is the appropriate regression analysis to conduct when the dependent variable is dichotomous (binary). Here we use logistic regression to compare the efficiency of TCW mutation and total mutation in predicting immunotherapy clinical response. Relationship between prognosis (patients with DCB or no DCB) and TCW mutation count or total mutation count was analyzed. The goodness of fit was performed by Hosmer–Lemeshow test (H-L test). The H-L test *P*-value of total mutation count is 0.0657 (Fig. 6b, left), close to 0.05, implicate the difference between prediction and expectation is close to significant. The H-L test *P*-value of TCW mutation count is 0.5791 (Fig. 6b, right), higher than the H-L test *P*-value of total mutation count, suggesting TCW mutation count is more suitable for predicting prognosis of patients than total mutation count. This analysis implicates APOBEC mutation count (represented by TCW mutation count) could be better than total mutation count in predicting immunotherapy response, and is in line with the observation that APOBEC mutational signature is specifically enriched in NSCLC patients with DCB after immunotherapy.

### APOBEC3B overexpression, *TP53* mutation and Nuclear Factor Kappa B (NF-κB) activation in immune regulation

To investigate the potential mechanism behind APOBEC-associated immune signature and immunotherapy response. We further studied the association between APOBEC3B expression and other common genetic alterations in NSCLC. *TP53* is a frequently mutated gene in NSCLC [29]. Recently it has been reported that *TP53* mutation is associated with immune gene expression signature, and can be served as an indicator of immune therapy response [30]. In breast cancer, it has been reported that APOBEC3B expression tends to lead to increased *TP53* mutation [31]. We found, similar to the situation in breast cancer, APOBEC3B expression is also associated with increased *TP53* mutations based on two independent NSCLC genomic databases (Supplementary Figures S4a and b). *TP53* mutation is also significantly associated with *APOBEC3B* mRNA expression but not CNV status (Supplementary Figures S4c and d). In addition, we observed that common NSCLC genetic alterations, *KRAS* and *STK11* mutations, are associated with downregulated APOBEC3B expression (Supplementary Figures S4a and b). This different mutation pattern may reflect the different stages when those mutations happen, as APOBEC-associated mutation tends to happen in late stage of cancer [5].

It is possible that APOBEC3B expression can directly lead to *TP53* mutation, or the selection of *TP53* mutated cells [31]. Wild-type TP53 was also reported to repress the transcription of APOBEC3B [32]. Inactivated TP53 can directly lead to NF-κB activation [33]. And NF-κB was reported to be able to stimulate the transcription of *APOBEC3B, PD-L1* and other immune response genes [34, 35]. In *TP53* mutated NSCLC, we did observe NF-κB activation and immune response signature (Supplementary Figure S5). *APOBEC3B* upregulation is significantly associated with both *TP53* mutation and NF-κB activation (Supplementary Figures S6a and b). Thus, APOBEC3B, TP53 and NF-κB may form an interconnected circuit in regulating immune response gene expression and consequently immunotherapy response (Supplementary Figure S6c).

## Discussion

APOBEC3B is known to play key roles in NSCLC mutagenic process, contributing to subclonal diversification, intra-tumor heterogeneity and tumor evolution [6]. APOBEC3B upregulation is also associated with poor NSCLC prognosis [15]. Here our study is focused on APOBEC3B. The function of other APOBEC3 family members in cancer is also been reported [36, 37]. The expression of some APOBEC3 family members (like APOBEC3A), but not others (like APOBEC3H) show similar expression pattern as APOBEC3B (Supplementary Figure 7a). Similar to APOBEC3B, overexpression of APOBEC3A, but not APOBEC3H predict poor NSCLC prognosis (Supplementary Figure 7b). Thus, APOBEC3 family members can play distinct and also overlapping function in NSCLC. Through combined cancer genomic mutation analysis and gene expression analysis, here we identified a significant correlation among *APOBEC3B* expression and immune gene expression, tumor immunotherapy response. Thus, our study suggests immunotherapy as a novel treatment option for APOBEC3B overexpression NSCLC.

Monoclonal antibodies against PD-1, PD-L1 yield significant clinical benefit for lung cancer patients by inhibiting immune checkpoint activity, but clinical predictors of response to these therapies remain incompletely characterized. Based on existing publications, the predictive markers for immune checkpoint inhibitor therapy include: PD-L1 expression [17, 18], tumor mutational load [19, 20], DNA mismatch repair (MMR) deficiency [21] and CD8^+^ T-cell intensity [22, 23]. We found APOBEC3B upregulation is positively associated with PD-L1 expression and immune cell infiltration, two known markers for immunotherapy response. Interestingly, a recent study also reported the association between PD-L1 expression and Kataegis mutational signature (caused by APOBEC), APOBEC3 expression [38]. Our results substantiate this observation, and additionally provide evidence to support the potential usage of APOBEC3B expression and APOBEC mutational signature as predictive biomarker for immunotherapy response.

Further study indicates that APOBEC mutational signature is specifically enriched in patients with good immunotherapy response, and APOBEC mutation count can be better than total mutation load in predicting immunotherapy response. As immune checkpoint blockade therapy is already in clinical use for NSCLC, APOBEC3B and APOBEC mutation count can be novel predictive markers for immune therapy response. DNA mismatch repair deficiency has recently been approved by FDA as the first tissue-agnostic biomarker for immunotherapy with pembrolizumab (anti-PD-1 antibody) [21]. APOBEC mutation is also widespread in human cancer patients, it is possible that APOBEC mutation in other types of cancer can also be served as biomarker for predicting immunotherapy response.

Based on association study, APOBEC3B, TP53 and NF-κB form an interconnected circuit in regulating immune gene expression and immunotherapy response in NSCLC. This hypothesis is in line with previous observation that TP53 mutation is a predictive marker for immune checkpoint blockade therapy response [30]. Besides PKC/NF-κB signaling, *APOBEC3B* expression was also reported to be induced by DNA-damaging drug [39] and DNA replication stress [40]. These DNA-damaging or stress signaling are known to stimulate *TP53* and select *TP53* mutation for cell to survival and proliferation.

Genetic alterations in somatic cell genomic DNA are major driving forces for cancer. Precision medicine based on individual cancer genome information show huge promise for future cancer treatment [41]. Previous reports imply that the sheer number of somatic aberrations could trigger an immune response, there was also report showing that total mutation load is not sufficient or not accurate for prediction of immune gene expression, and specific types of mutation can be more effective than others in doing so [42]. For tumor immunotherapy response, total mutation load was also reported to be not sufficient and accurate for predicting immunotherapy response in melanoma [43]. Some types of mutation could be better markers than total mutation load in predicting immunotherapy response. Our study implicates for the first time that APOBEC mutational signature can be a predictive marker for checkpoint blockade immunotherapy response. In together, our study not only suggests a novel therapeutic option for so far difficult to treat APOBEC3B-overexpressing NSCLC, but also identifies novel predictive markers for immunotherapy response.

## Materials and methods

### Clinical cohorts and cancer cell lines

The Cancer Genome Atlas (TCGA) NSCLC datasets include mRNA expression profiling (IlluminaHiSeq pancan normalized), gistic2 copy number, gene mutation, somatic mutation and patient prognosis information of 515 lung adenocarcinoma samples and 502 lung squamous cell carcinoma. GSE72094 cohort contains 442 patients with detailed mRNA expression data and EGFR/KRAS/TP53/STK11 Sanger sequencing data [44]. CCLE includes 187 lung cancer cell lines with detailed mRNA expression data [45]. The units of gene expression, copy number and protein expression of TCGA-NSCLC dataset are pan-cancer normalized log2(norm_count + 1), Gistic2 copy number and normalized RPPA value, respectively. The unit of gene expression of GSE72094 and CCLE datasets is log2 normalized RNA expression.

### Clinical samples, lung cancer cell lines sort and analysis of genes differential expression

For TCGA-NSCLC and GSE72094 mRNA expression data, the patients of *APOBEC3B* mRNA expression above the third quartile were defined as APOBEC3B UP group and the patients of *APOBEC3B* mRNA expression below the first quartile were defined as APOBEC3B DOWN group. Similarly in CCLE mRNA expression data, the lung cancer cell lines of *APOBEC3B* mRNA expression above the third quartile were defined as APOBEC3B UP group and the lung cancer cell lines of *APOBEC3B* mRNA expression below the first quartile were defined as APOBEC3B DOWN group. Sample sort, analysis of genes differential expression and heat map were done by using MeV (Multiple Experiment Viewer) software (http://mev.tm4.org/#/welcome).

### Gene set enrichment analysis

Pathways analysis was performed using the GSEA molecular signatures database (MSigDB) hallmark gene sets [46]. The query returned the top 10 gene sets showing significant enrichment. Color bar shading from light green to black, where lighter colors indicate more significant FDR *q*-values (<0.05) and black indicates less significant FDR *q*-values (≥0.05). FDR, false discovery rate; q value, minimum FDR at which the test was significant.

### mRNA expression profiling and reverse phase protein array (RPPA) analysis

For NSCLC samples included in the TCGA cohort, experimental procedures regarding tumor RNA extraction, mRNA library preparation, sequencing, quality control, and subsequent data processing for quantification of gene expression have been previously reported [29]. Gene expression data for the GSE72094 lung adenocarcinomas was obtained from GEO repository (http://www.ncbi.nlm.nih.gov/geo/query/acc.cgi?acc = GSE72094). mRNA expression of GSE72094 tumors were profiled using a custom Affymetrix GeneChip. mRNA expression data for lung cancer cell lines was obtained from the CCLE [45]. Protein expression was based on RPPA from TCGA database. The RPPA methodology and data analysis have been previously described [29].

### APOBEC mutational signature analysis

TCGA-NSCLC level 3 mutation data were downloaded from http://gdac.broadinstitute.org/. APOBEC mutagenesis signature was analyzed by using “P-MACD” R packages [36, 47]. APOBECs deaminate cytidines predominantly in a TCW motif (W = A or T). The APOBEC mutagenesis signature is composed of approximately equal numbers of two kinds of changes in this motif: TCW → TTW and TCW → TGW mutations. The analysis calculates the enrichment of the APOBEC mutational signature among all mutated cytosines in comparison with the fraction of cytosines that occur in the TCW motif among ±20 nucleotides surrounding each mutated cytosine on a per sample basis, and is described in detail in Roberts et al. [13]. APOBEC_enrich mean the enrichment over random of APOBEC pattern mutations. This is calculated as: {[(TCW to TGW) + (TCW to TTW)]/[(C to G) + (C to T)]}/[TCW/C]. The enrichment value > 2, which implies that in such samples at least 50% of APOBEC signature mutations have been in fact made by APOBEC enzyme(s). APOBEC_MutLoad_MinEstimate is the minimum estimate of number of APOBEC induced mutations in a sample. This estimate is calculated using the formula: [TCW to TGW + TCW to TTW]×[(APOBEC_enrich-1)/APOBEC_enrich] to determine the number of APOBEC signature mutations in excess of what would be expected by random mutagenesis. Calculated values are rounded to the nearest whole number.

### Mutational signature analysis

The mutational signature analysis was done by using latest R packages “SignatureAnalyzer” [48]. Somatic mutations in cancer genomes are caused by cumulative actions of several mutagenic processes that operate over the patient’s lifetime, including exposure to exogenous DNA-damaging agents or carcinogens (tobacco smoking or UV radiation), endogenous mutagens (reactive oxygen species or AID/APOBEC cytidine deaminases), or genomic defects in DNA repair or replicative processes [49, 50]. Non-NMF algorithm has been widely used in deciphering mutations signatures in cancer somatic mutations stratified by 96 base substitutions in trinucleotide sequence contexts. “SignatureAnalyzer” exploits a Bayesian variant of NMF algorithm to extract mutational signatures [48].

### Immune cell infiltration analysis

The abundance of immune cell infiltration was inferred by deconvolution approach TIMER [24], which can accurately resolves relative fractions of diverse cell subsets based on gene expression profiles from complex tissues. Only the relative fractions of CD8^+^ T cells are shown here. Another method called CIBERSORT was used to validate the results. CIBERSORT is an analytical tool developed by Newman et al. to provide an estimation of the abundances of cell types in a mixed cell population using gene expression data [51].

### Cancer immunotherapy dataset

Clinical and mutation data for 34 NSCLC patients were retrieved from cbioPortal (http://www.cbioportal.org/study.do?cancer_study_id = luad_mskcc_2015). These patients were treated with pembrolizumab (anti-PD-1 antibody) followed the protocol NCT01295827 (KEYNOTE-001). Overall, 14 patients show DCB (partial or stable response lasting >6 months), and 17 patients show NDB after treatment, the remaining 3 patients did not reach 6 months follow-up [20].

### Statistical analyses

Data between two groups were compared using a two-tailed unpaired Student’s *t*-test or Wilcoxon rank-sum test (also known as ‘Mann–Whitney’ test) depending on normality of data distribution (Typically, preprocessed expression data are normally distributed and mutation data show non-normal distribution). The lower and upper hinges of boxplot correspond to the first and third quartiles (the 25th and 75th percentiles). Notches are used to compare groups; if the notches of two boxes do not overlap, this suggests that the medians are significantly different. The upper whisker extends from the hinge to the largest value no further than 1.5 × IQR from the hinge (where IQR is the interquartile range, or distance between the first and third quartiles). The lower whisker extends from the hinge to the smallest value at most 1.5 × IQR of the hinge. Data beyond the end of the whiskers are called “outlying” points. All reported *P*-values are two tailed, and for all analyses, *P* ≤ 0.05 is considered statistically significant, unless otherwise specified. Multiple testing *P*-values were corrected by Benjamini–Hochberg method. Kaplan–Meier curves of overall survival were compared using the log-rank test. The goodness-of-fit for logistic regression was performed by H-L test using R package “ResourceSelection”. Small H-L test *P*-values mean that the model is a poor fit.

## Electronic supplementary material

Supplementary Figure legends

Figure S1

Figure S2

Figure S3

Figure S3-cont

Figure S4

Figure S5

Figure S6

Figure S7
