# Supplementary material for: APOBEC3B and APOBEC mutational signature as potential predictive markers for immunotherapy response in non-small cell lung cancer
Source: Oncogene. 2018 Apr 26;37(29):3924–36. doi: 10.1038/s41388-018-0245-9 (PMC6053356; doi:10.1038/s41388-018-0245-9)
Supplement: Supplementary file 1 — Supplementary Figure legends [file 41388_2018_245_MOESM1_ESM.docx]

**Supplementary Figure Legends**

**Figure S1.** APOBEC3B mRNA expression associated gene sets. Samples with *APOBEC3B* mRNA expression above the third quartile were defined as APOBEC3B-UP group and the patients of *APOBEC3B* mRNA expression below the first quartile were defined as APOBEC3B-DOWN group. (**a** and **b**) mRNA expression was compared between APOBEC3B UP and APOBEC3B DOWN NSCLC samples (**a**) or lung cancer cell line dataset (**b**), and genes with expression changes more than 0.5 fold were further analyzed by GSEA with MsigDB database. Significantly enriched top 10 gene sets are shown.

**Figure S2**. Recurrence quantification of mutational signature enrichment analysis. 100 independent simulations were performed with “SignatureAnalyzer” to assess the reliability of mutational signature analysis. The bar plots show that 3 mutational signatures (left panel) are recurrently enriched in DCB patients in every 100 independent simulations. While in NDB patients, 28 simulations enrich one mutational signature (W2, see Figure 5), 72 simulations enrich two mutational signatures (W1 and W2, see Figure 5) (right panel). Horizontal axis represents the number of signatures, while vertical axis represents frequency of generated signature after 100 independent simulations.

**Figure S3**. Mutational signatures and associations with immune gene expression in NSCLC. (**a**) Mutational signature analysis was performed with 1000 NSCLC samples with matched exome sequencing data, the specifically enriched signatures are shown. (**b**) Cosine similarity analysis of the identified mutational signatures, W4 signature is similar to COSMIC mutational signature 2 and 13, which are known to be caused by APOBEC. (**c**) The average mRNA expression (Log2 normalized count) of genes (n=60) from a TIL specific RNA-signature (reference 28) was used to rank the NSCLC samples. The vertical red line indicates the first quartile border. Top panel is the heatmap of gene expression, genes are in rows, samples in columns. Below the heatmap: the first row shows the average expression of the immune genes. In the remaining six rows, the length of bars are proportional to the number of substitutions of the indicated signatures. (**d**) The association between mutational signatures and average immune gene expression were statistically analyzed. For each types of mutational signature, a mutation cutoff was selected based on the distribution of mutation count. Number of samples with mutation number more than the cutoff are counted, and compared in left 25% and right 75% groups with Fisher's exact test. Only signature W4 (caused by APOBEC) show weak significant (P=0.04656) association in this analysis.

**Figure S4**. *TP53* mutation associated gene sets. mRNA expression was compared between *TP53* mutated and *TP53* wild type NSCLC samples, and genes with expression changes more than 0.5 fold were further analyzed by GSEA with MsigDB database. Significantly enriched top 10 gene sets are shown.

**Figure S5.** *APOBEC3B* expression is associated with *TP53* and *EGFR*, but not *KRAS* and *STK11* mutation based on two different datasets. (**a** and **b**) Percentage of common NSCLC mutations (*TP53, KRAS, EGFR* and *STK11*) between APOBEC3B UP and DOWN expression status in NSCLC patients based on the analysis of The Cancer Genome Atlas (TCGA) (**a**) and GEO repository (GSE72094) (**b**) database. (**c** and **d**) Significant up-regulation of *APOBEC3B* mRNA (**c**) but not CNV (**d**) in *TP53* mutated but not un-mutated NSCLC samples.

**Figure S6.** Potential interconnections among APOBEC3B, TP53 and NF-κB in regulating immune gene expression and immunotherapy response. (**a**) Correlations among *APOBEC3B* mRNA expression, TP53, phospho-p65 and phospho-PKC-alpha protein expression in NSCLC patients based on TCGA dataset. (**b**) Correlations between *APOBEC3B* mRNA expression and NF-kB-downstream target genes mRNA expression in NSCLC patients based on TCGA dataset. (**c**) Interconnections among *APOBEC3B* expression, *TP53* mutation and NF-κB activation in regulating tumor immune gene expression and immunotherapy response.

**Figure S7**. APOBEC3 family members’ expression and association with patient prognosis in NSCLC. (**a**) mRNA expression Heatmap of indicated APOBEC3 family members in APOBEC3B-UP and DOWN group. (**b**) Kaplan-Meier overall survival analysis based on *APOBEC3A* (top four plots), *APOBEC3B* (middle four plots) and *APOBEC3H* (bottom four plots) expression in four stages of NSCLC.
