# Supplementary material for: APOBEC3B and APOBEC mutational signature as potential predictive markers for immunotherapy response in non-small cell lung cancer
Source: Oncogene. 2018 Apr 26;37(29):3924–36. doi: 10.1038/s41388-018-0245-9 (PMC6053356; doi:10.1038/s41388-018-0245-9)
Supplement: Supplementary file 2 — Figure S1 [file 41388_2018_245_MOESM2_ESM.pdf]

Supplementary Figure 1

a

(TCGA) APOBEC3B associated pathways

Gene Set Name [# Genes (K)]	Description	# Genes in Overlap (k)	k/K	p-value	FDR q-value
HALLMARK_E2F_TARGETS [200]	Genes encoding cell cycle related targets of E2F transcription factors.	99		2.19 e^{-87}	1.09 e^{-85}
HALLMARK_G2M_CHECKPOINT [200]	Genes involved in the G2/M checkpoint, as in progression through the cell division cycle.	87		1.07 e^{-70}	2.67 e^{-69}
HALLMARK_INTERFERON_GAMMA_RESPONSE [200]	Genes up-regulated in response to IFNG [GeneID=3458].	56		8.24 e^{-34}	1.37 e^{-32}
HALLMARK_SPERMATOGENESIS [135]	Genes up-regulated during production of male gametes (sperm), as in spermatogenesis.	37		1.85 e^{-22}	2.32 e^{-21}
HALLMARK_ALLOGRAFT_REJECTION [200]	Genes up-regulated during transplant rejection.	42		1.8 e^{-20}	1.8 e^{-19}
HALLMARK_INTERFERON_ALPHA_RESPONSE [97]	Genes up-regulated in response to alpha interferon proteins.	30		3.82 e^{-20}	3.18 e^{-19}
HALLMARK_INFLAMMATORY_RESPONSE [200]	Genes defining inflammatory response.	40		9.19 e^{-19}	6.56 e^{-18}
HALLMARK_MTORC1_SIGNALING [200]	Genes up-regulated through activation of mTORC1 complex.	38		4.13 e^{-17}	2.58 e^{-16}
HALLMARK_KRAS_SIGNALING_UP [200]	Genes up-regulated by KRAS activation.	37		2.64 e^{-16}	1.32 e^{-15}
HALLMARK_MITOTIC_SPINDLE [200]	Genes important for mitotic spindle assembly.	37		2.64 e^{-16}	1.32 e^{-15}

b

(CCLE) APOBEC3B associated pathways

Gene Set Name [# Genes (K)]	Description	# Genes in Overlap (k)	k/K	p-value	FDR q-value
HALLMARK_TNFA_SIGNALING_VIA_NFKB [200]	Genes regulated by NF-kB in response to TNF [GeneID=7124].	93		7.24 e^{-87}	3.62 e^{-85}
HALLMARK_INTERFERON_GAMMA_RESPONSE [200]	Genes up-regulated in response to IFNG [GeneID=3458].	86		1.09 e^{-76}	2.73 e^{-75}
HALLMARK_INTERFERON_ALPHA_RESPONSE [97]	Genes up-regulated in response to alpha interferon proteins.	52		3.14 e^{-53}	5.24 e^{-52}
HALLMARK_EPITHELIAL_MESENCHYMAL_TRANSITION [200]	Genes defining epithelial-mesenchymal transition, as in wound healing, fibrosis and metastasis.	68		1.32 e^{-52}	1.65 e^{-51}
HALLMARK_INFLAMMATORY_RESPONSE [200]	Genes defining inflammatory response.	67		2.33 e^{-51}	2.33 e^{-50}
HALLMARK_HYPOXIA [200]	Genes up-regulated in response to low oxygen levels (hypoxia).	56		2.5 e^{-38}	2.08 e^{-37}
HALLMARK_COMPLEMENT [200]	Genes encoding components of the complement system, which is part of the innate immune system.	54		4.25 e^{-36}	3.03 e^{-35}
HALLMARK_IL2_STAT5_SIGNALING [200]	Genes up-regulated by STAT5 in response to IL2 stimulation.	51		7.74 e^{-33}	4.84 e^{-32}
HALLMARK_KRAS_SIGNALING_UP [200]	Genes up-regulated by KRAS activation.	49		1.01 e^{-30}	5.62 e^{-30}
HALLMARK_APOPTOSIS [161]	Genes mediating programmed cell death (apoptosis) by activation of caspases.	44		6.63 e^{-30}	3.32 e^{-29}
