# Supplementary figures and images for: APOBEC3B and APOBEC mutational signature as potential predictive markers for immunotherapy response in non-small cell lung cancer
Source: Oncogene. 2018 Apr 26;37(29):3924–36. doi: 10.1038/s41388-018-0245-9 (PMC6053356; doi:10.1038/s41388-018-0245-9)

Supplementary Figure 2

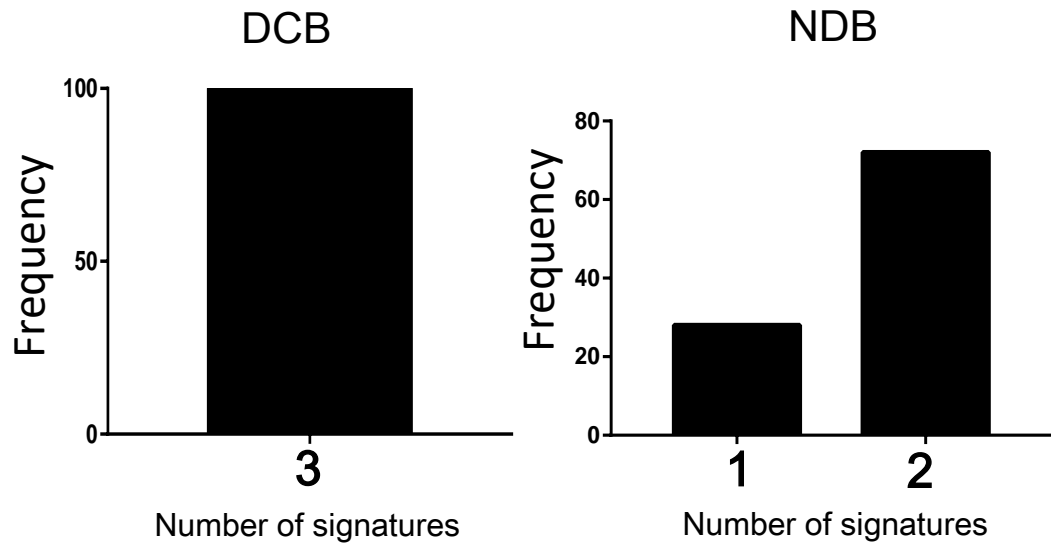

Supplement: Supplementary file 3 — Figure S2 [file 41388_2018_245_MOESM3_ESM.pdf]

# Supplementary Figure 3

**a**

Mutation Signatures in TCGA NSCLC

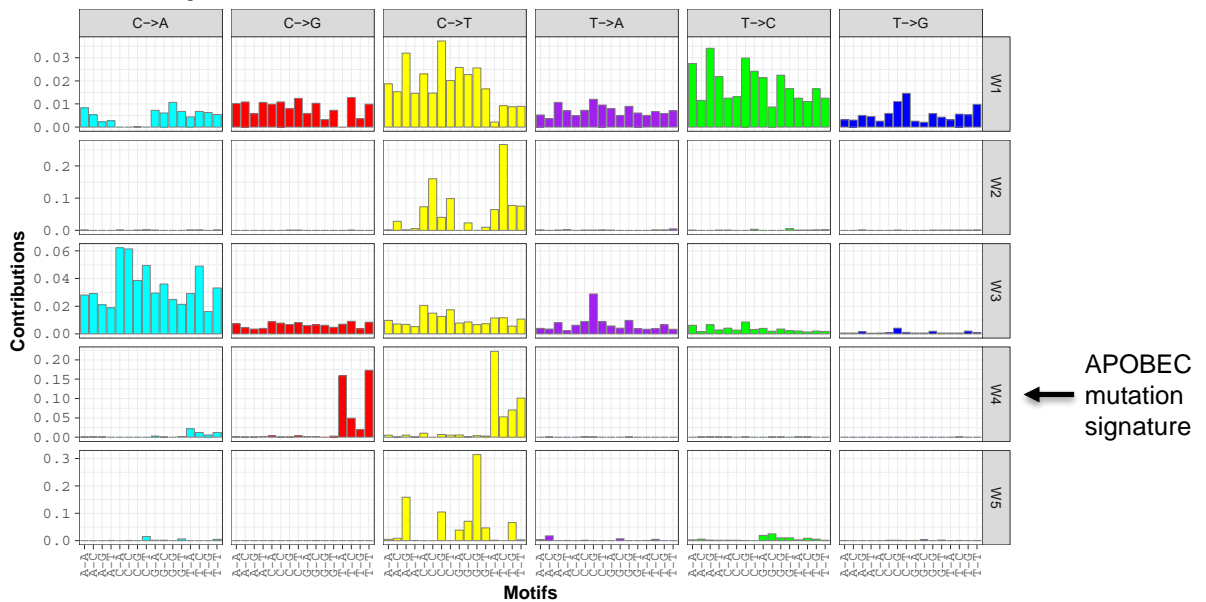

**b**

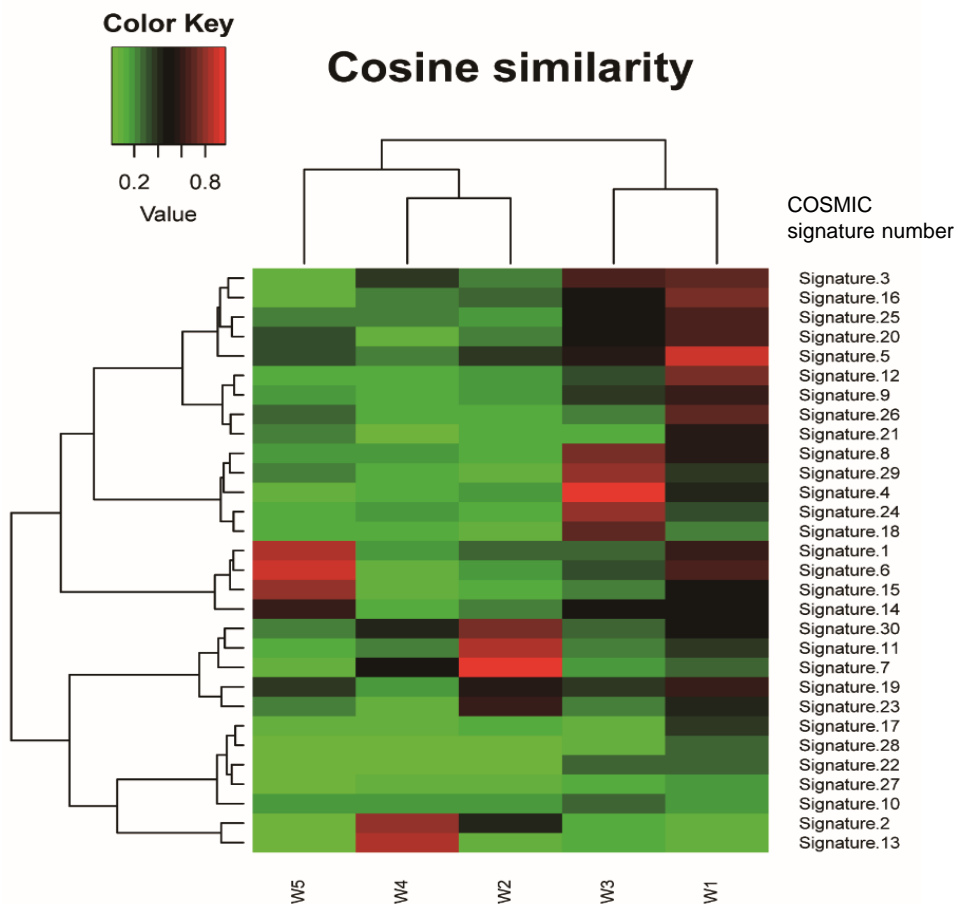

Supplement: Supplementary file 4 — Figure S3 [file 41388_2018_245_MOESM4_ESM.pdf]

# Supplementary Figure 4

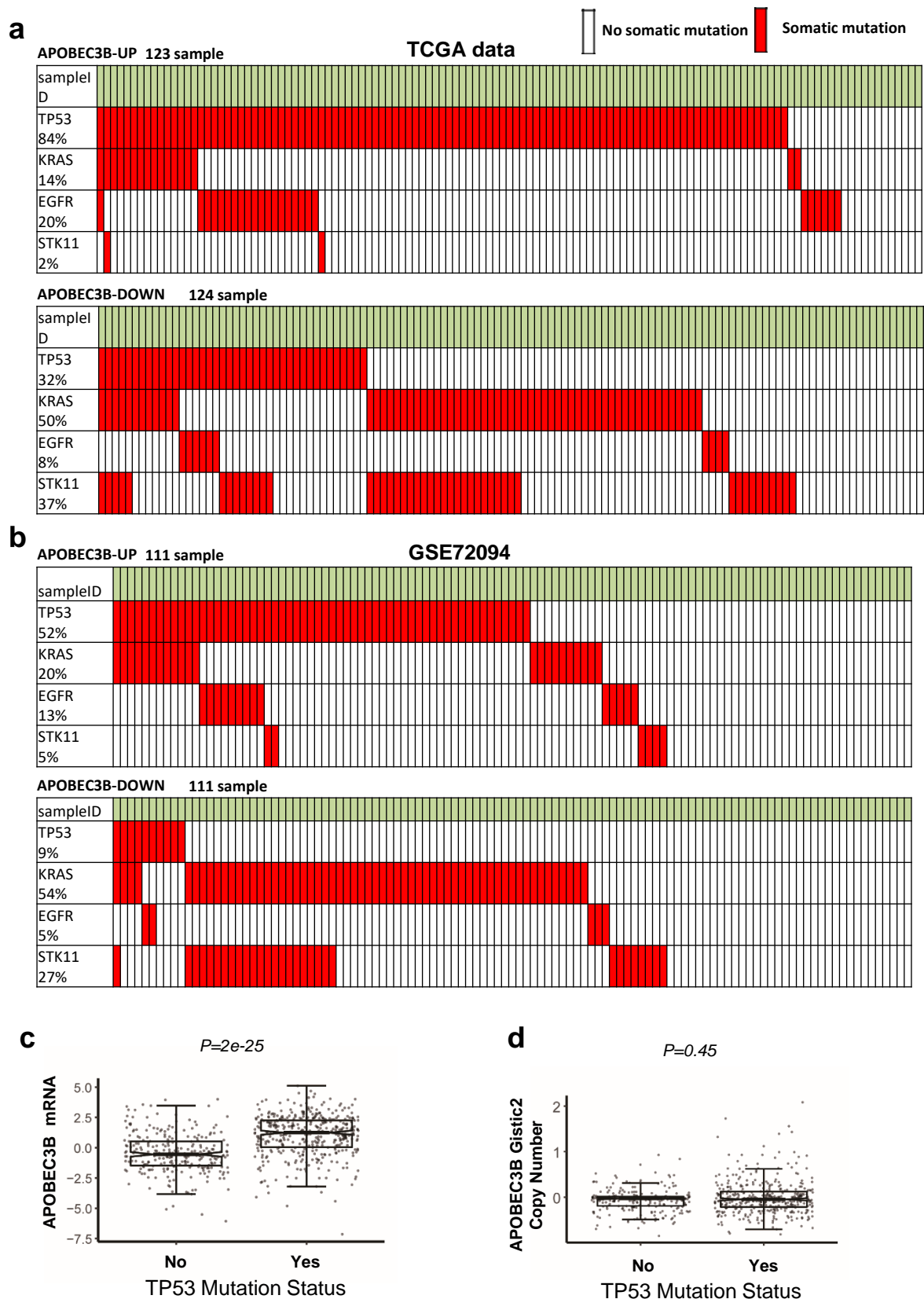

Supplement: Supplementary file 6 — Figure S4 [file 41388_2018_245_MOESM6_ESM.pdf]

## Supplementary Figure 6

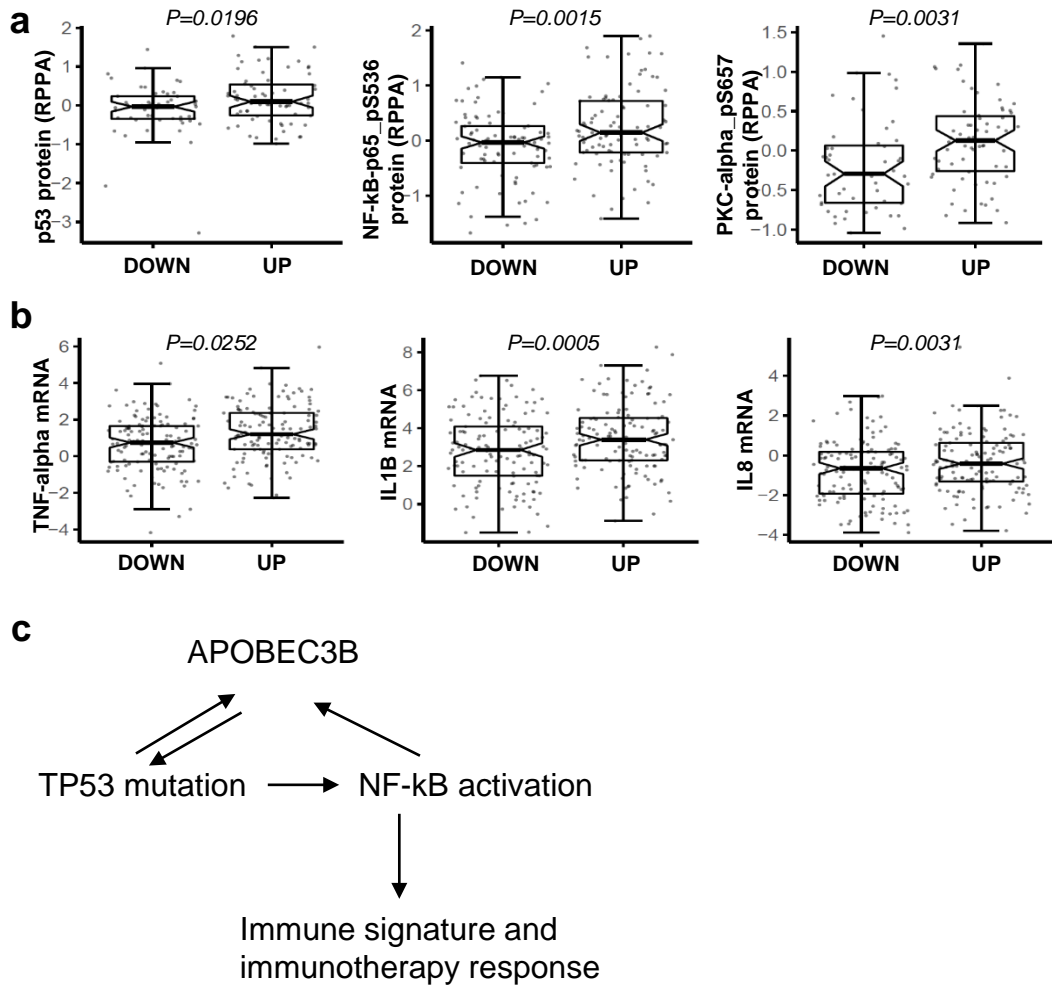

Supplement: Supplementary file 8 — Figure S6 [file 41388_2018_245_MOESM8_ESM.pdf]

# Supplementary Figure 7

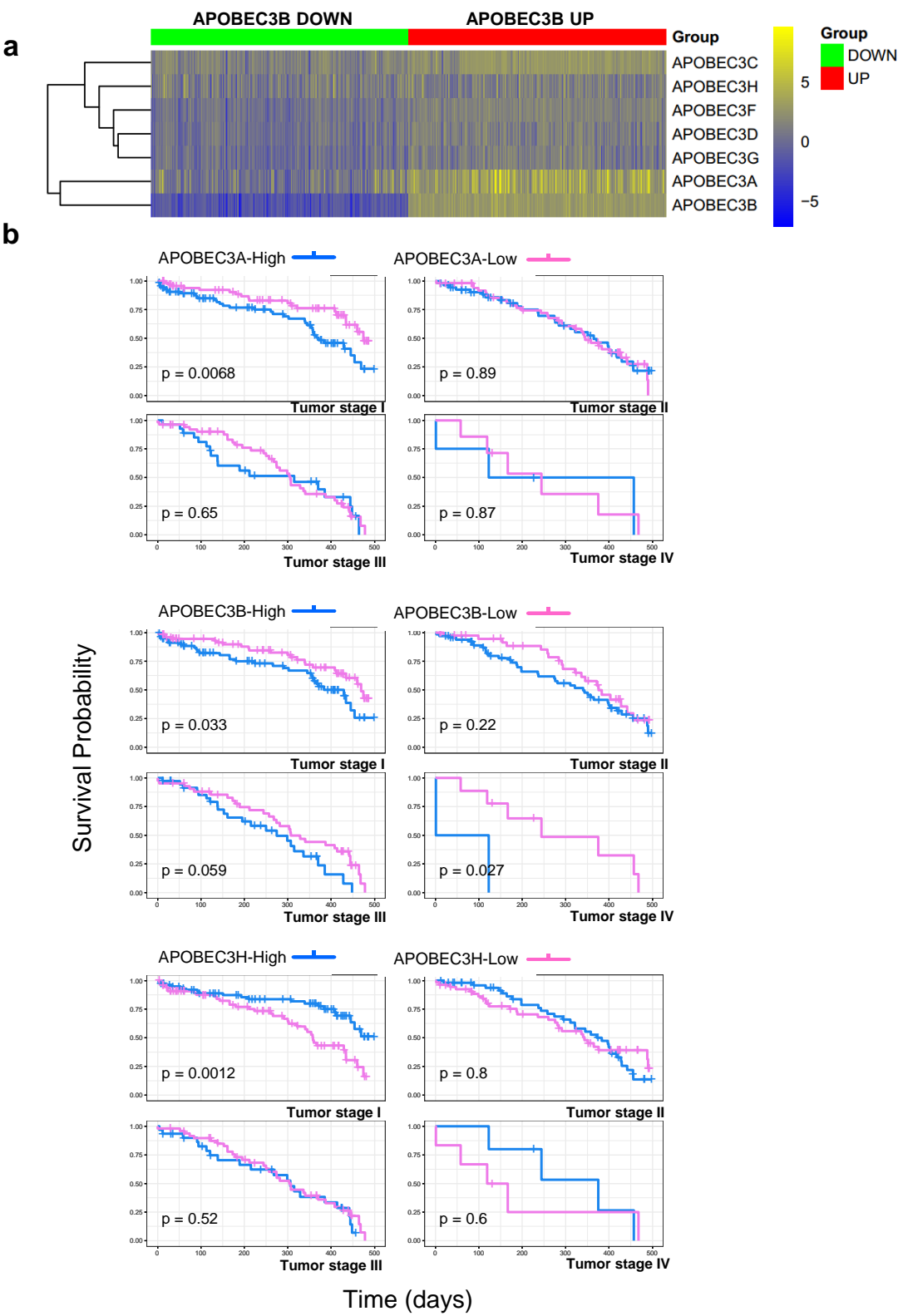

Supplement: Supplementary file 9 — Figure S7 [file 41388_2018_245_MOESM9_ESM.pdf]
