# Supplementary material for: APOBEC3B and APOBEC mutational signature as potential predictive markers for immunotherapy response in non-small cell lung cancer
Source: Oncogene. 2018 Apr 26;37(29):3924–36. doi: 10.1038/s41388-018-0245-9 (PMC6053356; doi:10.1038/s41388-018-0245-9)
Supplement: Supplementary file 5 — Figure S3-cont [file 41388_2018_245_MOESM5_ESM.pdf]

Supplementary Figure 3

c

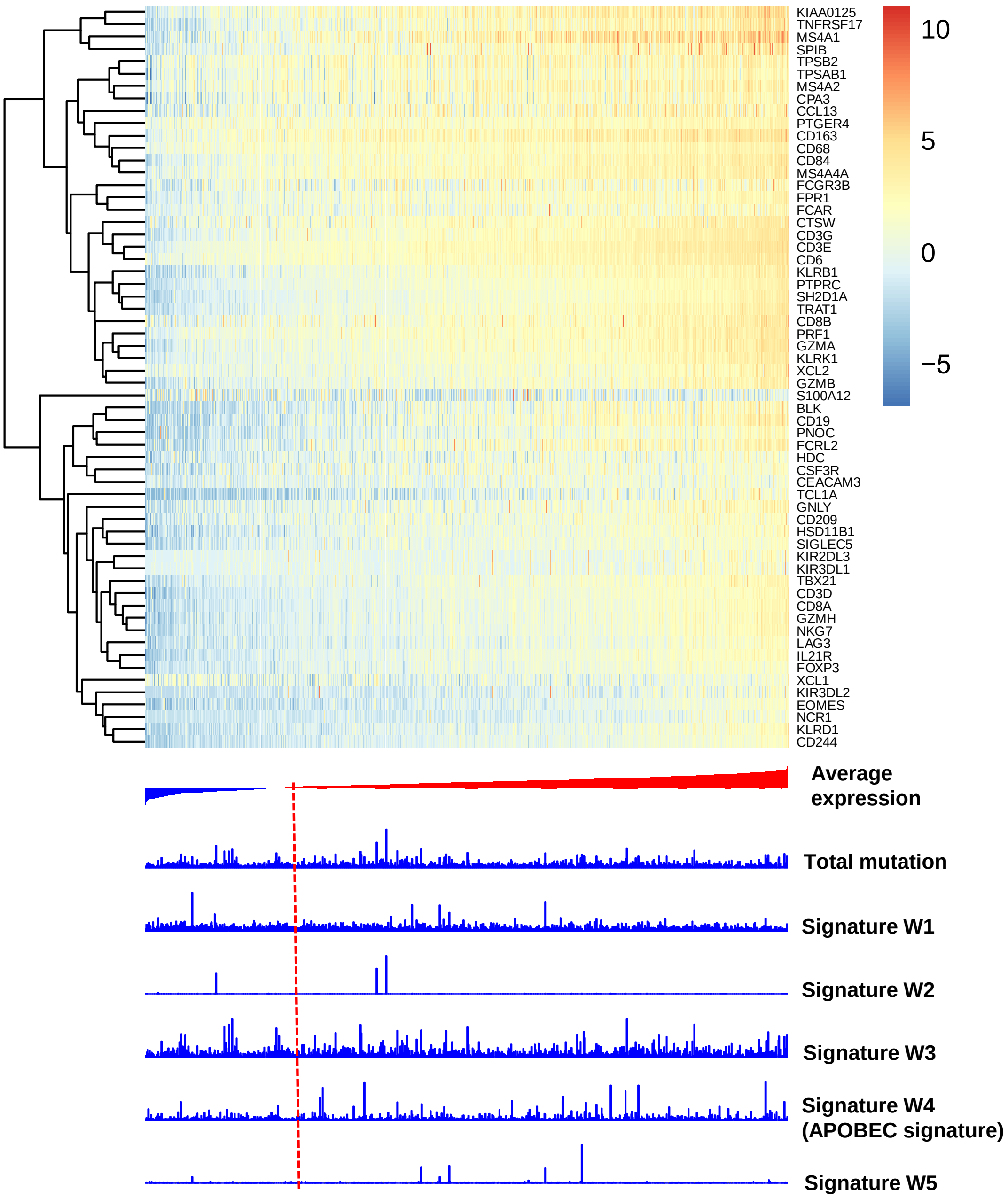

d

Signature	Fisher exact test p value	Higher mutation samples in Left 25%	Higher mutation samples in right 75%	Mutation cutoff	Corresponding COSMIC signature number
W1	0.4386	11	25	150	5
W2	0.1048	2	3	60	7,11
W3	0.7812	17	57	562	4
W4	0.04656	5	38	176	2,13
W5	0.6873	1	7	43	6,1
Total Mutation	0.7168	9	33	1210	NA
