# Supplementary material for: APOBEC3B and APOBEC mutational signature as potential predictive markers for immunotherapy response in non-small cell lung cancer
Source: Oncogene. 2018 Apr 26;37(29):3924–36. doi: 10.1038/s41388-018-0245-9 (PMC6053356; doi:10.1038/s41388-018-0245-9)
Supplement: Supplementary file 7 — Figure S5 [file 41388_2018_245_MOESM7_ESM.pdf]

Supplementary Figure 5

(TCGA) TP53 mutation associated pathways

Gene Set Name [# Genes (K)]	Description	# Genes in Overlap (k)	k/K	p-value	FDR q-value
HALLMARK_INTERFERON_ALPHA_RESPONSE [97]	Genes up-regulated in response to alpha interferon proteins.	13		$3.1\ e^{-14}$	$1.55\ e^{-12}$
HALLMARK_INTERFERON_GAMMA_RESPONSE [200]	Genes up-regulated in response to IFNG [GeneID=3458].	15		$1.86\ e^{-12}$	$4.66\ e^{-11}$
HALLMARK_INFLAMMATORY_RESPONSE [200]	Genes defining inflammatory response.	12		$4.05\ e^{-9}$	$6.76\ e^{-8}$
HALLMARK_E2F_TARGETS [200]	Genes encoding cell cycle related targets of E2F transcription factors.	10		$4.42\ e^{-7}$	$5.53\ e^{-6}$
HALLMARK_G2M_CHECKPOINT [200]	Genes involved in the G2/M checkpoint, as in progression through the cell division cycle.	9		$3.98\ e^{-6}$	$3.98\ e^{-5}$
HALLMARK_KRAS_SIGNALING_UP [200]	Genes up-regulated by KRAS activation.	8		$3.2\ e^{-5}$	$2\ e^{-4}$
HALLMARK_MITOTIC_SPINDLE [200]	Genes important for mitotic spindle assembly.	8		$3.2\ e^{-5}$	$2\ e^{-4}$
HALLMARK_TNFA_SIGNALING_VIA_NFKB [200]	Genes regulated by NF-kB in response to TNF [GeneID=7124].	8		$3.2\ e^{-5}$	$2\ e^{-4}$
HALLMARK_SPERMATOGENESIS [135]	Genes up-regulated during production of male gametes (sperm), as in spermatogenesis.	6		$1.78\ e^{-4}$	$9.47\ e^{-4}$
HALLMARK_IL6_JAK_STAT3_SIGNALING [87]	Genes up-regulated by IL6 [GeneID=3569] via STAT3 [GeneID=6774], e.g., during acute phase response.	5		$1.89\ e^{-4}$	$9.47\ e^{-4}$
